# Quantifying the tumour vasculature environment from CD-31 immunohistochemistry images of breast cancer using deep learning based semantic segmentation

**DOI:** 10.1186/s13058-024-01950-2

**Published:** 2025-02-04

**Authors:** Tristan Whitmarsh, Wei Cope, Julia Carmona-Bozo, Roido Manavaki, Stephen-John Sammut, Ramona Woitek, Elena Provenzano, Emma L. Brown, Sarah E. Bohndiek, Ferdia A. Gallagher, Carlos Caldas, Fiona J. Gilbert, Florian Markowetz

**Affiliations:** 1 Institute of Astronomy, University of Cambridge, Cambridge, UK; 2 Cancer Research UK Cambridge Institute, University of Cambridge, Cambridge, UK; 3 School of Medicine, University of California San Francisco, San Francisco, US; 4 Department of Radiology, University of Cambridge, Cambridge, UK; 5 Breast Cancer Now Toby Robins Research Centre, The Institute of Cancer Research, London, UK; 6 The Royal Marsden Hospital NHS Foundation Trust, London, UK; 7 Research Center for Medical Image Analysis and Artificial Intelligence (MIAAI), Danube Private University, Krems, Austria; 8 Cambridge University Hospitals NHS Foundation Trust, Cambridge, UK; 9 Cancer Research UK Beatson Institute, University of Glasgow, Glasgow, UK; 10 Department of Clinical Biochemistry, University of Cambridge, Cambridge, UK; 11 Institute of Metabolic Science, University of Cambridge, Cambridge, UK

## Abstract

**Background:**

Tumour vascular density assessed from CD-31 immunohistochemistry (IHC) images has previously been shown to have prognostic value in breast cancer. Current methods to measure vascular density, however, are time-consuming, suffer from high inter-observer variability and are limited in describing the complex tumour vasculature morphometry.

**Methods:**

We propose a method for automatically measuring a range of vascular parameters from CD-31 IHC images, which together provide a detailed description of the vasculature morphology. We first used a U-Net based convolutional neural network, trained and validated using 36 partially annotated whole slide images from 27 patients, to segment vessel structures and tumour regions from which the measurements are taken. The model also segments the vascular smooth muscle, benign epithelium, adipose tissue, stroma, lymphocyte clusters, nerves and CD-31 positive leukocytes, and we applied it to an additional 21 images from 15 patients. Using these segmentations, we investigated the relationship between the various tissue types and the vasculature and studied the relationship of various vascular parameters with clinical parameters. We also performed a 3D histology analysis on a separate tumour sample as a proof of principle, providing a more comprehensive visualization of vasculature morphology compared to the standard 2D cross-section of a tissue sample.

**Results:**

Using two-way cross-validation, we show that vessels were accurately segmented, with Dice scores of 0.875 and 0.856, and were accurately identified, with F1 scores of 0.777 and 0.748. All vascular parameters exhibit strong ($r>0.7$) and significant (*p*<0.001) correlations with measurements taken from the manual ground truth vessel segmentations. A significant relationship between the major/minor axis ratio, a measure of elongation, and the tumour grade was found.

**Conclusion:**

Our proposed method shows promise as a tool for studying the tumour vasculature and its relationship with surrounding cells and tissue types. Furthermore, the correlation with tumour grade highlights the clinical relevance of our approach. These findings suggest that our method could have substantial implications for improving prognostic assessments and personalizing therapeutic strategies in breast cancer treatment.

## Introduction

 The vasculature plays a key role in the tumour micro-environment by providing the necessary nutrients for the growth and survival of cancer cells [1]. Vascular endothelial growth factor (VEGF) produced by tumours induce angiogenesis. The resulting rapid neo-vascularisation results in a greater number and more immature vessels, characterised by a reduced pericyte layer. An impaired pericyte coverage results in less functional and more leaky vessels. Together with the growth of the tumour this creates a high interstitial fluid pressure, causing the vessel lumen to narrow or collapse [2, 3]. The rapid tumour growth leads to a disorganised vascular network, with tortuous vessels and blunt ends. The resulting reduced blood flow limits the amount of oxygen perfusion and diffusion within the tumour. The cancer cells become hypoxic leading to a more aggressive type of a tumour, which are more resistant to therapeutics. Furthermore, hypoxia-inducible factor (HIF) causes an up-regulation of VEGF, making this an aggregated process [4].

Breast cancer can be highly vascularised, making this process of development of hypoxia prominent [5]. Although various techniques for imaging the breast tumour vasculature have been studied [6], immunohistochemistry (IHC) remains the gold standard. In particular the use of CD-31, which is a highly specific marker for endothelial cells, is common in the examination of the vasculature in histopathology.

Since manually counting all vessels on CD-31 IHC slides is time consuming, estimation methods have been developed, and attempts have been made to standardise these estimations [7]. Although such metrics of vascular density have been shown to be of prognostic value [8], they are not used in clinical practice due to the subjective nature of these measurements [9], as well as being time consuming and of limited descriptiveness. Automatic methods [10–13] or semi-automatic approaches [14] have been previously developed to address this problem. However, they may have limited performance when applied to breast cancer, where CD-31 IHC can lead to a considerable amount of false positive staining of tissue other than vessels. Specifically, the expression of CD-31 in macrophages [15] and plasmacytic/plasmablastic lesions [16] have been identified as a potential for misdiagnosis. Furthermore, while simple colour-based stain identification methods may perform well for basic tasks like vessel counting, they fall short for more advanced quantification tasks. These methods often struggle to accurately measure complex vascular features because common vascular markers typically stain only the endothelial cells in the vessel wall. This frequently results in incomplete representations, such as hollow centers within vessels or fragmented vessel walls, particularly when the stain does not bind uniformly or when parts of the vessel wall are missing due to tissue sectioning. Thus, there is a need for a more robust segmentation method that provides a more detailed and accurate depiction of the tumour vasculature.

All this highlights the need for a comprehensive assessment of the tumour vascular microenvironment. New advances in deep learning provide a solution to this by learning the imaging features that distinguish one cell or tissue type from the other. Thus, we use a deep learning-based approach to automatically segment tissue structures and quantify the tumour vascular microenvironment on full slide IHC images. With the CD-31 images being counter-stained with hematoxylin to show the cell nuclei, we are able to identify tumour regions and distinguish them from benign epithelial cells, as well as other distinct tissue types such as nerves, smooth muscle and lymphocyte clusters. We subsequently propose various parameters of the tumour vasculature extracted from the resulting segmentations, and report the segmentation performance with respect to ground truth manual segmentations. With several regional analyses we explore the relationship of the vasculature and lymphocytes with respect to the tumour, stroma and adipose tissue. Finally, we investigate the potential clinical value of the proposed method by relating the various vascular and tissue measurements with the tumour grade and node status. We also performed a 3D histology analysis on a separate tumour sample to gain further insights.

## Materials and methods

### Data

**Fig. 1:**
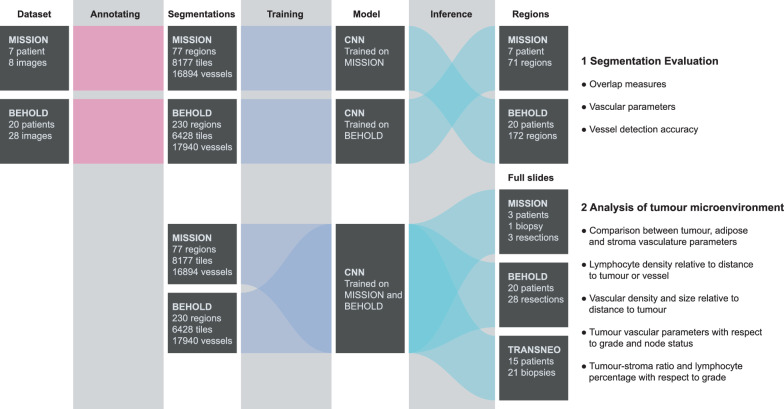
A flowchart visualization of the study design, with the top (Section 1) showing the annotation, training, and cross-validation of the Convolutional Neural Network (CNN). The bottom (Section 2) illustrates the setup for the analysis of the tumour microenvironment in the 38 ER-positive patients using a model constructed from the entire annotated dataset. In this section we specify how many of whole slide images are from a needle biopsy or a resection, which represents a larger tissue section removed during surgery

Three datasets, each from a different clinical study: MISSION [National Research Ethics Service Committee East of England, Cambridge South, Research Ethics Committee number (REC) 15/EE/0378; National Institute for Health Research (NIHR) portfolio number 30388], BEHOLD [REC: 14/EE/0145] and TRANSNEO [REC: 12/EE/0484], were retrospectively analysed in this study. They include core biopsies and resections from patients with a range of breast cancer types. The complete dataset includes 8 whole slide images from 7 patients in the MISSION study, 28 images from 20 patients in the BEHOLD study, and 21 images from 15 patients in the TRANSNEO study. Note that multiple images from the same patient may originate from the same sample or from different samples taken from different locations. The TRANSNEO dataset became available later in the project and was included to increase the overall dataset size, increasing the robustness of our findings. The occurrence of each tumour type, the number of ER-, PR-, and HER2-positive patients, and the grades are provided for each dataset in Table 1. Digital images were acquired at 20x magnification resulting in images with a pixel size of 0.5034 × 0.5034 µm. These datasets are illustrated in Fig. 1. Written, informed consent was provided by all study participants.

**Table 1:** Pathology occurrence for each dataset

	Behold	Mission	Transneo
	(n = 20)	(n = 7)	(n = 15)
Invasive Ductal Carcinoma	15	5	14
Ductal Carcinoma in situ	14	0	0
Invasive Mucinous Carcinoma	1	0	0
Invasive Lobular Carcinoma	5	1	1
Lobular Carcinoma in situ	3	0	0
Invasive Spindle Cell Metaplastic Carcinoma	0	1	0
ER-positive	20	3	15
PR-positive	19	2	8*
HER2-positive	5	0	8
Grade 1	3	0	0
Grade 2	8	1	6
Grade 3	9	6	9

n = number of patients. * PR-status not available for 7 patients

An additional dataset of an ER-patient-derived xenograft (PDX) was collected for 3D histology examination. A cryopreserved breast PDX tumour fragment ( 2 mm^3^) in freezing media (foetal bovine serum, heat-activated Thermo Fisher Scientific 10500064 +10% dimethyl sulfoxide Sigma D2650) was defrosted at 37 °C, washed with Dulbecco’s modified eagle’s medium (Gibco 41966) and mixed with matrigel (Corning 354262) before surgical implantation. Tumours were implanted subcutaneously into the flank of 6–9 week-old NOD SCID gamma (NSG) mice (Jax Stock #005557) as per standard protocols.

**Fig. 2:**
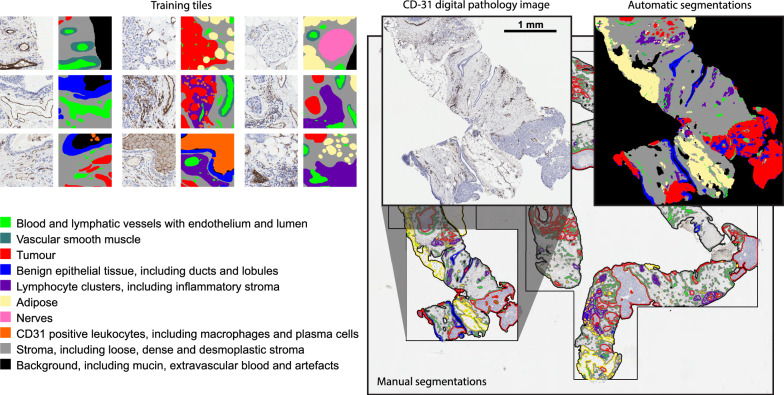
Left: Representative selection of tiles (512 × 512 pixels) with the corresponding tissue labels by manual annotation used for training the deep learning models. Right: Manual and automatic segmentation of the full CD-31 IHC digital pathology slide of a core needle biopsy from the MISSION study using a CNN trained on the BEHOLD study data

Manual annotations of the various tissue types were performed by the first author using the brush tool in QuPath v0.2.0-m2 [17]. For the biopsies the entire tissue section was used (Fig. 2), while for the resections regions were selected to attempt to capture the full range of variation present in the slide (Supplementary Figure S1). The tissue classes include vessels, vascular smooth muscle, tumour regions, benign epithelial cells, adipose, lymphocyte clusters, CD-31 positive leukocytes, nerves and background. The remaining tissue is subsequently classified as stroma.

A convolutional neural network (CNN), as described below, was subsequently trained on this data and was used to segment an additional set of regions. These regions were manually corrected, again using the brush tool in QuPath, to form a final large annotated dataset to be used for the training of the various models in this study. All segmentations were verified and, if necessary, corrected by a trained pathologist specialised in breast pathology. Various background regions with image artefacts were also selected to be used only during training, to increase the robustness of the final model when doing full slide inference.

To train and evaluate the deep learning model, the binary maps for each class were exported and converted into tiles of 512x512 pixels. Figure 2 provides some representative samples with all of the tissue types.

### Deep learning architecture

The deep learning architecture, as well as the training, inference, data processing, and evaluation routines, including statistical analysis, were all implemented in Python (version 3.7). In line with a data-centric AI approach, we use a standard U-Net architecture [18], implemented in Keras (version 2.4.0) and Tensorflow (version 2.4.4), with minimal modifications. Compared to the original U-Net, we incorporate batch normalisation and use LeakyReLu as the activation function to prevent the “dying ReLU” problem. Each convolution, is preceded by a reflection padding. This will avoid the progressive shrinkage of the feature map. In addition we use mixed precision which saves memory and allows us to use a batch size of 23 using an NVIDIA Tesla V100S 32GB GPU.

For data augmentation we use a random flip, rotation with mirror padding, as well as color jitter. The weights were optimised using Adam with an exponentially decaying learning rate and warm restarts. As the cost function we use a weighted soft Dice loss defined as:

1 $$\begin{aligned} Loss(p,g) = 1 - \sum _{j}^{C} \frac{w_j}{ \sum _l^C w_l } \cdot \frac{2\sum _{i}^{N}p_{i,j} \cdot g_{i,j} }{\sum _{i}^{N}p_{i,j}+\sum _{i}^{N}g_{i,j}} \end{aligned}$$

where $p\in [0, 1]$ denotes the prediction probability, and $g\in \left\{ 0,1 \right\}$ the ground truth at the *i*-th pixel of the *j*-th class, with the set of pixels *N* and classes *C*. The weights of each class are defined by *w* and estimated based on their prevalence and importance. Thus, with adipose, stroma and background being rather prevalent they are given a weight of 1. tumour, benign tissue, lymphocytes and muscle are given a weight of 2 while macrophages and nerves, being rather rare, are given a value of 5. Considering the segmentation performance of the blood vessels is of prime importance, it is given a weight of 10.

### Inference and post-processing

The final layer in the CNN is a Softmax function which provides a probability map for each class. The inference was done using a sliding window approach across the entire slide, whereby the probabilities of each class are generated in 512 × 512 regions with an overlap of 128 pixels. The probability maps were weighted based on the linear distance to the center of tile and the binary segmentations for each class were subsequently derived from them using majority voting.

The following post processing operations were applied:

1. A gaussian smoothing was applied to the probability maps of all tissue classes except the vessels and leukocyte structures to retain the level of detail in them.
2. A hole filling algorithm was applied to the background on regions smaller than 10000 pixels.
3. A hole filling algorithm was applied to the stroma on regions smaller than 1000 pixels overwriting all but the vessel and leukocyte structures.
4. A hole filling algorithm was applied to the adipose on regions smaller than 10000 pixels overwriting all but the vessel and leukocyte structures.
5. A hole filling algorithm was applied to the vessel segmentations overwriting any other classes.

### Quantification

Several parameters were extracted from the resulting vascular segmentation maps using custom code. These parameters are illustrated in Supplementary Figure S2 and explained below:

- *Density* (#/mm^2^): The number of vessels per square mm within the stroma of the region of interest.
- *Mean area* (µm^2^): The mean of the surface areas of the blood vessels.
- *Mean circularity*: (Circularity = $(4 \cdot  \pi \cdot Area) / Perimeter^2)$The area versus perimeter ratio, which is 1 for a perfect circle and decreases with complexity down to 0.
- *Mean axis ratio*: The mean of the ratio between the long and short edge of the rectangles fitted over each vessel segmentation.
- *Mean thickness* (µm): The mean of the vessel thicknesses, measured using a maximum diameter sphere fitting algorithm.

For the average axis ratio, a minimum bounding box was fitted over each vessel segmentation. The ratio of the long edge over the short edge defines the major/minor axis ratio of the vessel and the average ratio was subsequently calculated over all vessels in the image or region. The vessel thickness was derived from a measure developed for quantifying the trabecular bone thickness [19]. A distance map was first generated from the segmentation mask, as well as a skeleton mask. For each point in the skeleton mask a sphere was drawn with a radius and value corresponding to the value from the distance map at this location. Pixel values were only overwritten when the new value is greater than the old values. The thickness was then calculated as the average pixel value in the thickness map within the vessel segmentation.

## Results

This study is composed of two parts as depicted in Fig. 1 and outlined below, as well as an additional 3D analysis.

### Segmentation validation

The evaluation of the U-Net based segmentation performance of the tissue regions and vessels, as well as the measurements of the vascular parameters were performed in a two way cross-validation split based on the MISSION or BEHOLD study.

The manually annotated regions and the vascular parameters extracted from the manually segmented vessels were considered the ground truth. The cross-validations were performed in such a way that the CNN is trained on the manually segmented data from one study and validated on the other. In Supplementary Figure S3 we can see the confusion matrix of the predictions versus true classes assessed on a pixel by pixel basis for every image segment.

The segmentation performance of the tissue regions was assessed using the Dice score and Jaccard index calculated over every image segment (Table 2). The performance of identifying individual vessels was also assessed, as reported in Table 3. We show a precision, recall and F1 score for the proposed method over baseline. Here we assume that an overlap between a predicted vessel and true vessel $>50\%$ with respect to the true vessel regions indicates a True Positive. Furthermore, we show how the predicted vessels might be inaccurately identified as more than one (number of splits) or several vessels are inaccurately identified as one vessel (number of merged) as percentage with respect to the total number of vessels.

**Table 2:** The segmentation performance measured by Dice score and Jaccard index for both datasets in the cross-validation

	Behold		Mission	
	Dice	Jaccard	Dice	Jaccard
Vessel	0.875	0.777	0.856	0.748
Tumour	0.701	0.539	0.815	0.688
Benign	0.476	0.312	0.556	0.385
Adipose	0.898	0.814	0.915	0.843
Lymphocytes	0.698	0.536	0.648	0.480
Muscle	0.714	0.555	0.736	0.582
Leukocytes	0.715	0.557	0.780	0.640
Nerve	0.764	0.618	0.873	0.775
Stroma	0.886	0.796	0.899	0.817
Background	0.753	0.604	0.954	0.913

**Table 3:** The vessel detection performance and percentage of splits and merges relative to the total number of vessels (n), measured for both datasets in the cross-validation

	Behold	Mission
	(n = 17940)	(n = 16894)
Precision	0.791	0.776
Recall	0.655	0.727
F1 score	0.717	0.750
merged (%)	2.386	3.344
split (%)	1.048	0.900

All vascular parameters measured from the validation set are correlated with the measurements taken from the ground truth manual segmentations for both partitions in the cross-validation. Here vessels smaller than 40 pixels (10 µm^2^) are considered noise and are discarded. Using Pearson’s correlation we show a strong correlation ($r>0.7$) for all parameters with all correlations being statistically significant ($p<0.001$). The correlation plots are shown in Supplementary Figure S4.

### Analysis of tumour vascular microenvironment

To investigate the function of the vasculature in the tumour microenvironment, we required full slide annotations of the available biopsies and resections. For this we again used a CNN to provide an automated full slide segmentation. In this case we built a single CNN from all data to ensure highly accurate segmentations. In addition we included a third dataset from the TRANSNEO study, which included diagnostic biopsies with corresponding clinical data. The resulting full slide annotations allowed us to do a detailed analysis of relationships between the vasculature parameters and other tissue regions or pathologies. In these analyses we excluded vessels smaller than 40 pixels and tumour or adipose regions smaller than 1000 pixels (250 µm^2^ ) to exclude possible artefacts or singular adipose or tumour cells. For this analysis we included data only from patients with ER-positive breast cancer since the ER status has been shown to be associated with characteristics of the vasculature [20].

**Fig. 3:**
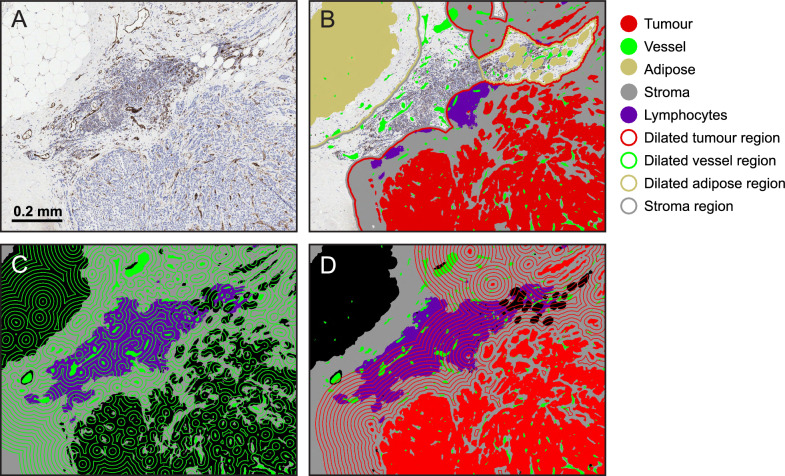
Examples of the regions used in the various examinations. The original IHC image (**A**). The tumour, adipose, and stroma regions (**B**), defined by dilating the tumour and adipose segmentations by 150 µm. The consecutive regions around the vessels (**C**), each corresponding to 25 µm. The consecutive regions around the tumour (**D**), each corresponding to 25 µm

**Fig. 4:**
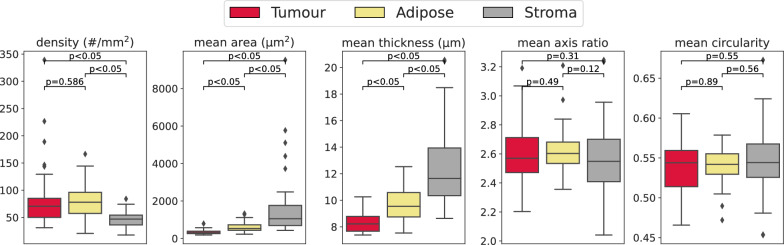
Comparison of the vasculature parameters in the tumour, adipose, and stroma regions for the 38 ER-positive patients, represented by box plots with outliers defined as values beyond 1.5 times the interquartile range. *P*-values by two-tailed, two-sample t-tests

We first attempted to verify the differences in the vascular parameters within stroma, adipose and tumour tissue. To define these regions, we first applied a morphological dilation of 150 µm to the tumour region and the adipose region, while ensuring no overlap between them. This was done by applying a smaller dilation in turn, while not overwriting the other region. The remaining stroma region was defined as including the lymphocyte clusters. These three regions subsequently defined the regions of interest for measuring the vascular parameters (Fig. 3A and B). A comparison of each parameter per patient with respect to the tissue region is provided in Fig. 4.

**Fig. 5:**
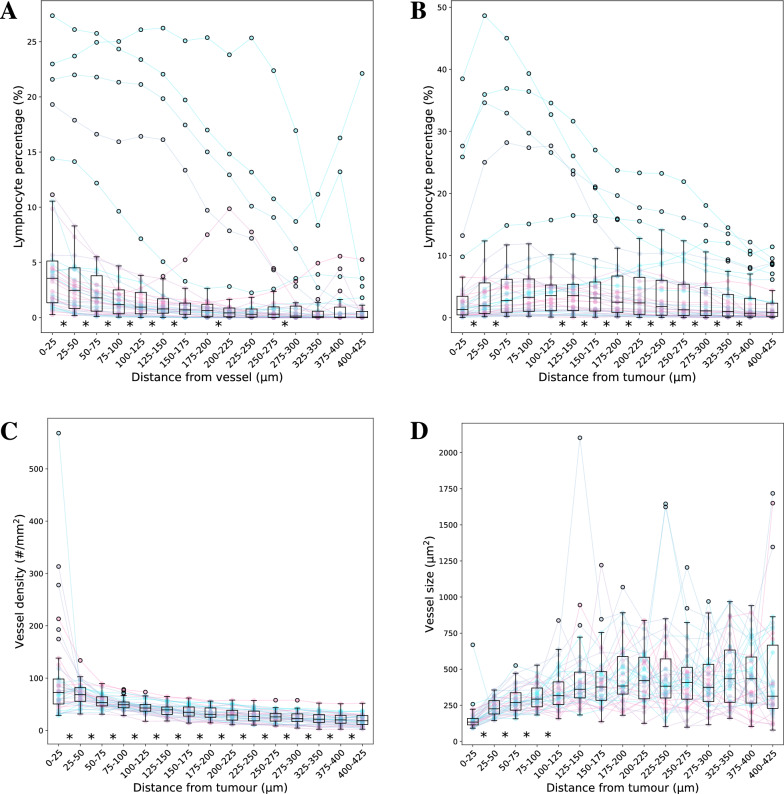
The lymphocyte density relative to the distance from the vessel (**A**) or tumour (**B**), and the vessel density (**C**) and size (**D**) relative to the distance from the tumour for the 38 ER-positive patients. The box plots display outliers as black circles beyond 1.5 times the interquartile range. Each randomly coloured dot represents an individual patient’s data for a specific distance range, and the same colour is used for each patient across all distance ranges, with lines connecting the dots to show the trend for that patient across the full range of distances. * $p<0.05$ between consecutive regions assessed using two-tailed, paired t-tests

To assess the relationship of the lymphocytes with respect to the vasculature and tumour cells, we performed a regional analysis. Starting with the vascular segmentations, we applied consecutive morphological dilations of 25 µm to extract regions corresponding to a range of distances to the vessel (Fig. 3C). For each region we subsequently calculate the lymphocyte percentage, which is defined by the lymphocyte area relative to the combined stroma and lymphocyte area. The same was done for the tumour whereby the lymphocyte percentage was assessed for consecutive regions around the tumour (Fig. 3D). The subsequent plots are shown in Fig. 5A and B. Paired t-tests between the consecutive regions show that the lymphocytes are more prominent in close proximity to the vessels. This suggested a strong relationship between the lymphocytes and the vasculature. Furthermore, the lymphocyte percentage (lymphocyte density) decreased when measuring further away from the tumour. However, this was only the case when measuring more than 150 µm from the tumour border. The majority of lymphocytes appeared around the 125–150 µm distance from the tumour, while reducing significantly when measuring close to the tumour border.

To investigate this further, a similar regional analysis was performed for the vessel density and size with respect to the distance from the tumour (Fig. 5C and D). Vessels closer to the tumour boundary were more numerous and smaller. This corresponds to the hypothesis that the tumour incurs a rapid neovascularisation.

**Fig. 6:**
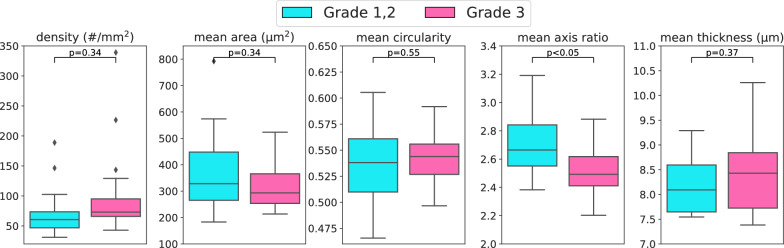
Bar plot of vascular parameters against grade for the 38 ER-positive patients, with outliers defined as values beyond 1.5 times the interquartile range.* P*-values by two-tailed, two-sample t-tests with Benjamini-Hochberg correction

To understand the significance of the vascular measurements with respect to clinical parameters, we perform several additional evaluations. We compare the tumour vasculature parameters as described previously between patients with grade 1 or 2 and those with grade 3 (Fig. 6). Here a significant difference was seen in the mean axis ratio with respect to the tumour grade. In addition, the parameters were compared with respect to the lymph node status where available (Supplementary Figure S5), but this showed no significant differences.

Within the tumour regions we can also measure the tumour-stroma ratio, which gives us a quantitative measure of the tumour morphology. Here the stroma is defined as including the lymphocyte regions. In the same way the lymphocyte percentage within the tumour region provides a quantitative measure of the tumour infiltrating lymphocytes. These measures were again evaluated with respect to the Grade (Supplementary Figure S6). No significant differences were observed here.

### Extension to 3D

**Fig. 7:**
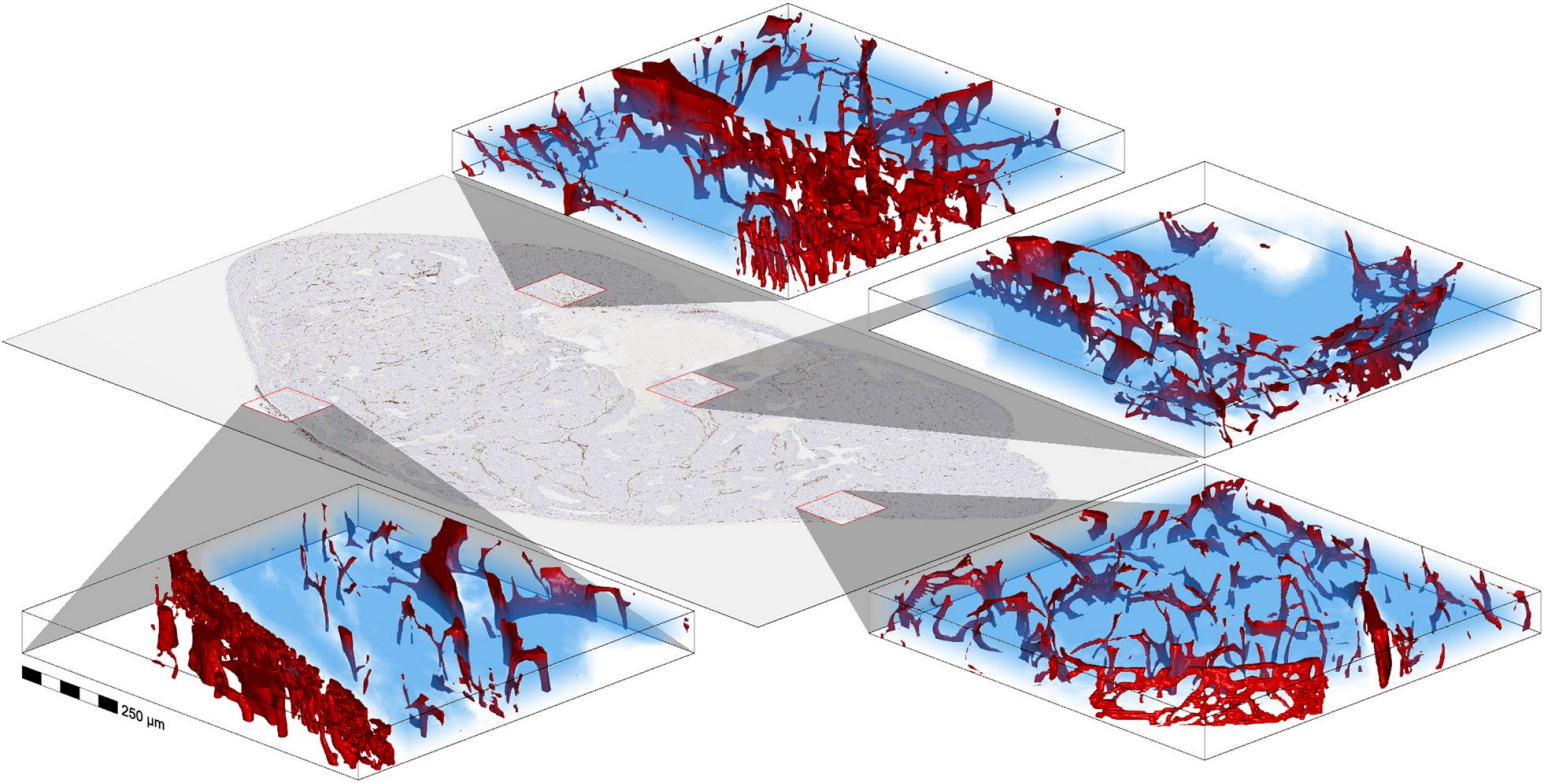
3D histology of four regions in a human tumour grown as a xenograft in a recipient mouse. The segmented tumour is shown as a transparent blue volume, while the segmented vessels are shown as a red surface mesh

Now that we have an automatic vessel segmentation tool, we are also able to perform a segmentation of the vasculature in 3D by using serial section histology. For this we use 22 serial sections of CD-31 IHC images of a human tumour grown as a xenograft in an immunodeficiency mouse. The serial sections were first aligned and the deformations corrected using a custom module in ScanXm^1^ (Minogame Ltd, Cambridge, UK). The deep learning based segmentation method was subsequently applied to each section to generate 3D tumour and vessel segmentations. A surface mesh was then generated for each vessel structure, which is shown together with a volume rendering of the tumour segmentation in Fig. 7. For each volume also an animation is provided in the supplementary videos. In Supplementary video S1 we can see normal vessels at the tumour borders, which may contain some skin vessels. However, when examining the vessels inside of the tumour, they appear highly disorganised (Supplementary video S2). In some parts they appear to be regular vessels that were compressed by a growing tumour or high interstitial fluid pressure to become flat latices (Supplementary video S3). Interestingly, at the border of the necrotic region we also see regular vessels (Supplementary video S4), which may have appeared in response to an increased VEGF, while not being suppressed by the tumour.

## Discussion

In this study we proposed a deep learning based method for the automatic segmentation and subsequent quantification of the tumour vasculature environment from CD-31 IHC images.

Our vessel segmentation performance, with a Dice score of 0.875 and 0.856, and a Jaccard index of 0.777 and 0.748, is comparable to previous studies which segment the vasculature from H&E digital pathology images. Yi et al. [21] report a Jaccard index of 0.755, while Frazz et al. report a Dice score and Jaccard index of 0.9390 and 0.8851 in [22], and 0.8714 and 0.7721 in [23]. These studies, however, use standard H&E stained slides, which have some considerable limitations compared to the use of IHC markers specific to the vasculature. They rely heavily on red blood cells being present in the vessels, which might not be the case. Furthermore, H&E sections will only clearly show the larger vessels, while it is the micro-vasculature which is of particular interest in cancer biology. Illustrative of this is a study showing that the presence of vascular invasion is easily missed when assessed from H&E slides [24]. To acquire accurate measures of the vascular morphology, CD-31 IHC remains the most appropriate modality by highlighting the epithelial cells in the vessel wall, thereby allowing us to get a more complete picture of the microvasculature structure. A direct comparison of our segmentation results with the above papers will therefore not be appropriate.

We furthermore show that the proposed vascular parameters extracted from the vessel segmentations correlate well with the parameters measured from the ground truth manual segmentations, with strong ($r>0.7$) and significant ($p<0.001$) correlations for all parameters.

Other markers, such as Von Willebrand Factor (vWF) and CD34, are also used to highlight vasculature. However, CD31 is often preferred because it is generally more sensitive than vWF in detecting small blood vessels and capillaries. While CD34 is another commonly used vascular marker, it is less suitable for quantifying vessels in breast cancer due to its lower specificity, staining not only endothelial cells but also stromal and non-endothelial cells. Nonetheless, these markers may be appropriate in other contexts. Considering the high level of augmentation used in training the neural network, the same model may work just as well on these markers, although some fine-tuning with data specific to those stains may further improve its performance.

By examining the differences in vascular parameters relative to other tissue types we show that these parameters vary in expected ways. In [25], a higher vascular density had been measured in adipose-rich tissue compared to stroma-rich tissue in cancer-adjacent breast tissue. Figure 4 demonstrates that the vascular density, defined as the number of blood vessels per square millimetre within the stroma of the adipose region, is significantly greater ($p<0.05$) than the vascular density in the remaining stroma. This similarly suggests that the proximity of adipose tissue is associated with increased vascularisation within the stroma. In the same figure we also show that stroma vasculature near the tumour is more dense, as well as being of smaller size, compared to the vasculature in the stroma further away from the tumour. These findings are also in line with the common notion that neo-vascularisation occurs in proximity to the tumour. This was examined in greater detail in a regional analysis of vascular density and size relative to the distance from the tumour. In this analysis, we demonstrated that vessel density significantly decreases, and vessel size significantly increases as the measurement distance from the tumour border increases (Fig. 5C and D). All this gives confidence in the validity of these measurements.

Regarding the segmentation performance of tumour regions, we show a Dice score of 0.701 and 0.815. This is an improvement over previous studies, such as the by of Cruz-Roa et al. [26]. There, a Dice score of 0.6041 was reported for the segmentation of a mixture of invasive and noninvasive breast cancer, as is the case in our study. The difference in performance can be explained by the more detailed tumour delineation in our data. Importantly, these results show that tumour regions can be segmented from only the hematoxylin stained cell nuclei in IHC images. This eliminates the need for the co-registration with H&E images when examining structures seen in IHC in proximity to other tissue types.

In order for the tumour tissue to be classified correctly, it was necessary to also include benign epithelial tissue in the model to prevent this from being misclassified as tumour tissue. Its low segmentation performance is due to the way tumour regions were defined. Since we do not identify individual cells, the annotated tumour regions may contain a considerable number of normal cells. So when the neural network correctly predicts some cells as being normal, they may be within a region defined as tumour. Hence, the neural network should be better at correctly identifying benign tissue than what the Dice score of 0.476 and 0.556 indicates.

Also the lymphocyte regions are of limited segmentation performance, which is due to its loose definition as a cluster of lymphocytes. This may result in annotations that differ greatly in appearance between tissue samples. However, once the model is trained, it will provide unbiased predictions, thus generating consistent segmentations.

The nerve tissue has previously been identified as a possible structure of interest and has been included in a model to segment together with the vasculature from H&E images [23]. Using their proposed FABNet architecture they report a Dice score of 0.879 for the nerve segmentation, which is consistent with our Dice score of 0.764 and 0.873.

In our study the CD-31 positive leukocytes show a low segmentation performance, as the CD-31 positive macrophages and plasma cells are easily misclassified as a blood vessels [15, 16]. Although it is difficult to assess whether our vessel detection with an F1 score $>0.7$ (Table 3) is sufficiently accurate, by including these structures in the model, our proposed method may at least reduce the number of false positives.

In this study we have shown the potential value of vascular parameters in assessing the pathological status of the tumour. This is evident in the significant difference observed in the mean major/minor axis ratio relative to the tumour grade (Fig. 6). However, none of the other parameters have shown any relationship with the grade. This is despite the mean number of vessels having previously been shown to be inversely related to the tumour grade in breast cancer [27].

Previous research has established a link between lymphatic vessel density and lymph node metastasis [28]. Our analysis of the vascular density with respect to the node status shows no significant relationship. However, it is worth noting that prior to adjusting for multiple comparisons, the relationship approached significance (*p*=0.06). The use of CD-31, which highlights both lymphatic and blood vessel walls without differentiation, presents a challenge in this analysis. Removing the influence of blood vessels from this assessment, possibly through the use of a marker specific for the lymphatic endothelium such as D2-40 [24, 29], may improve the predictive value of this metric.

A low tumour to stroma ratio (TSR) has been shown to be associated with poor clinical outcomes in most solid tumour types [30], which includes triple negative breast cancer [20]. However, a recent study using an automated image analysis using QuPath shows that for ER-positive breast cancer the opposite is true, with a low TSR being associated with a favourable prognosis [31]. Our results corroborate these findings by showing a lower TSR for patients with a lower grade, although the differences are not statistically significant.

By measuring the lymphocyte percentage within the tumour stroma region we provide a pseudo measure for the amount of TILs, which have previously been shown to be associated with outcome [32–34]. In accordance with these previous studies we show that a greater lymphocytes percentage was associated with high grade, but again not statistically significant. In [35] a computational tumour infiltrating lymphocytes (cTILs) biomarker was proposed, which is substantially equivalent to our lymphocyte percentage measure. An ROC analysis showed that this biomarker achieved 100% sensitivity in predicting pathological complete response (pCR), although also not reaching statistical significance. In [36] graph-based features derived from paired tile-level classification maps was used to predict pCR and residual disease (RD) using machine learning methods. Here the tumour and TILs interaction was shown to best predict pCR, while the microvessel density and polyploid giant cancer cells pair was most predictive for RD. While all these studies are limited by their sample size, they do show the value of examining and quantifying the various cellular and tissue interactions within the tumour vascular microenvironment.

When examining the tumour tissue in 3D, the tumour vasculature appears complex and highly disorganized. The 2D descriptors proposed in this work may not be adequate for characterizing these intricate morphologies. Some recent work does indeed show a prognostic and predictive value in breast cancer by measuring the 3D vascular morphology from the larger vessels seen in CT and contrast-enhanced MRI [37].

Some limitations of this study should be noted. A complication encountered in our study is the presence of extravascular blood. Since red blood cells are often visible within blood vessels, the deep learning model may learn to identify a cluster of extravascular red blood cells as a blood vessel, thereby introducing an error to the vascular parameter measurements. Until a solution is developed for this, more care in the histological processing, or a manual removal of these regions will prevent this source of error.

Another limitation of this study is the limited number of images available to us due to the infrequent use of CD-31 IHC in clinical practice. Although in absolute numbers we use a substantial amount of annotated data, they were derived from relatively few samples, thereby potentially inducing a batch effect. Nonetheless, our study shows that the CD-31 IHC images provide a comprehensive view of the tumour microenvironment with potentially valuable insights. Integrating CD-31 IHC alongside standard H&E staining, while minimally disrupting the clinical workflow, may therefore become more prevalent in the future.

## Conclusion

In this study, we developed a method for automatically measuring parameters describing the vasculature morphology from CD-31 IHC images. By segmenting a range of tissue types, we were also able to examine the interactions of the vasculature with its environment. Furthermore, we have shown a significant relationship between the major/minor axis ratio and the tumour grade, suggesting future clinical utility of the proposed method. More work is required to assess whether the proposed or other, potentially three-dimensional, vascular measurements from digital pathology imaging have any prognostic or predictive value in clinical practice, or could inform future treatment decisions.

## Additional file

Supplementary file 1.

Supplementary file 2.

Supplementary file 3.

Supplementary file 4.

Supplementary file 5.

## Data Availability

The code used for this study is publicly available on GitHub at https://github.com/TristanWhitmarsh/quantifying-tumour-vasculature-environment. The data that support the findings of this study are available from the corresponding author, T.W., upon reasonable request.

## Footnotes

1 https://www.scanxm.com/
