# Supplementary material for: Quantifying the tumour vasculature environment from CD-31 immunohistochemistry images of breast cancer using deep learning based semantic segmentation
Source: Breast Cancer Res. 2025 Feb 4;27:17. doi: 10.1186/s13058-024-01950-2 (PMC11796191; doi:10.1186/s13058-024-01950-2)
Supplement: Supplementary file 1 [file 13058_2024_1950_MOESM1_ESM.pdf]

Supplementary data

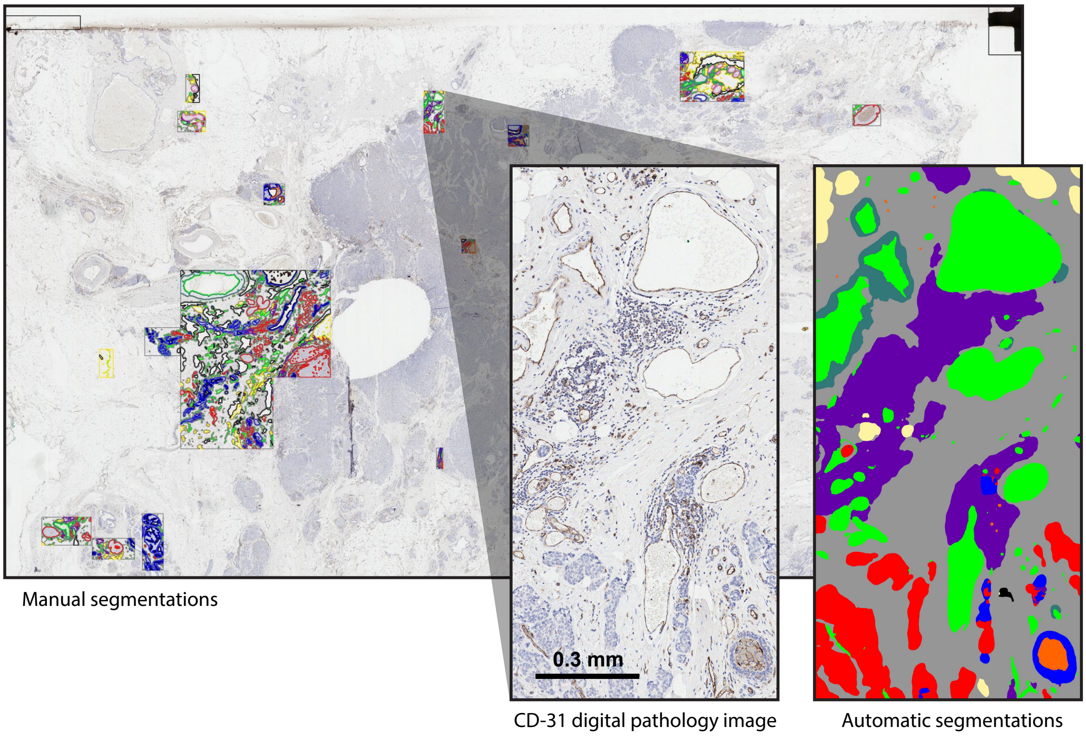

Supplementary Figure 1: Manual and automatic segmentation of various regions of a CD-31 digital pathology image of a resection from the MISSION study using a CNN trained on the BEHOLD study data.

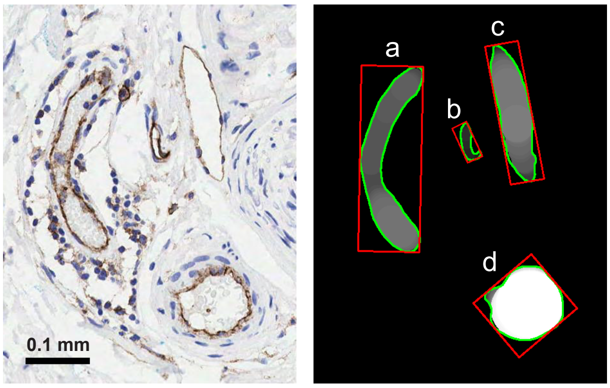

a
Area: 2954 μm^2
Circularity: 0.27
Axis ratio: 3.00
Thickness: 20.1 μm

b
Area: 237 μm^2
Circularity: 0.41
Axis ratio: 2.30
Thickness: 7.8 μm

c
Area: 2209 μm^2
Circularity: 0.44
Axis ratio: 4.08
Thickness: 23.1 μm

d
Area: 2623 μm^2
Circularity: 0.83
Axis ratio: 1.06
Thickness: 51.0 μm

Supplementary Figure 2: Illustration of the vascular measurements. The CD-31 IHC digital pathology image with four distinct vessels (left) and the thickness map represented by grayscale values (right). The vessel boundary is shown in green, which is used for the calculation of the circularity. The bounding box is shown in red, which is used for calculating the axis ratio. The values of the four vascular measurements are provided for each of the vessels.

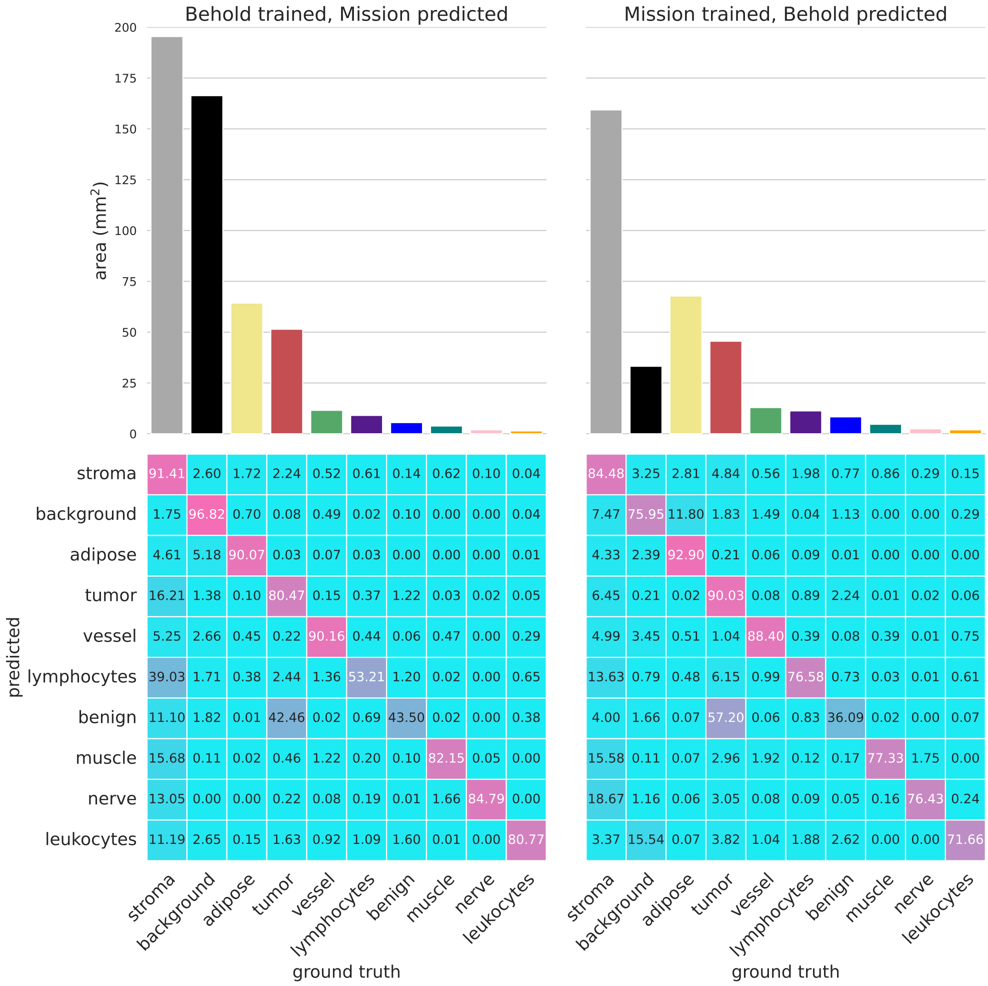

Supplementary Figure 3: The confusion matrix (bottom panels) for the model trained on the Behold data and tested on the Mission data (left) and trained on the Mission data and tested on the Behold data (right). The values in the confusion matrix have been normalised with respect to the ground truth (columns). The top panels show the size of the respective tissue types used in the evaluation.

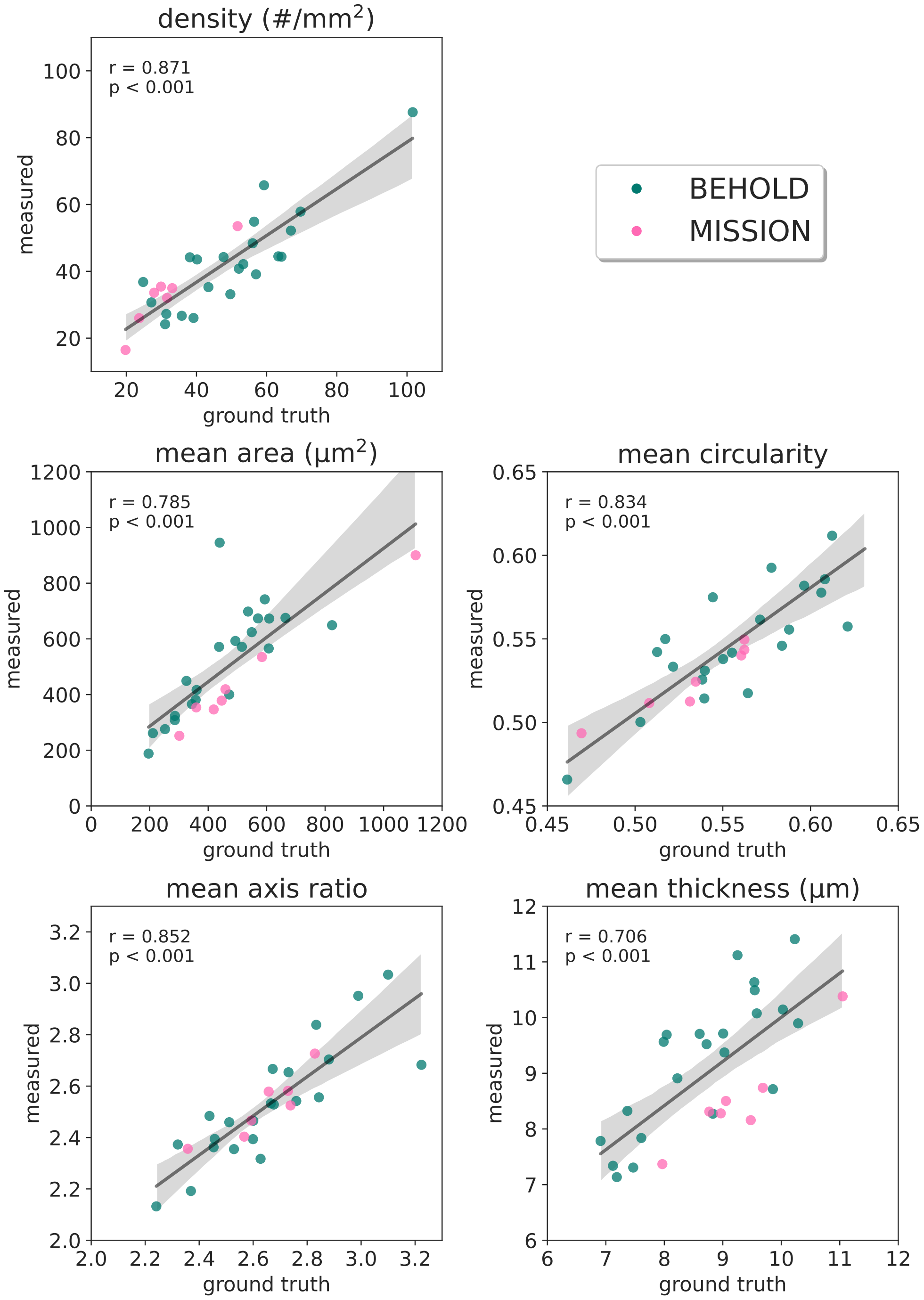

Supplementary Figure 4: Scatter plots with linear correlations and 95% confidence intervals of the various vascular parameters as well as the Pearson correlation coefficients (r) with associated p-values.

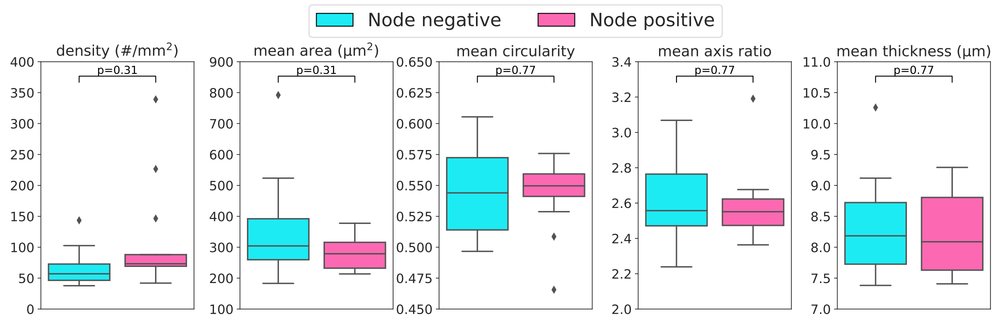

Supplementary Figure 5: Bar plot of vascular parameters against node status for 31 ER positive patients, with outliers defined as values beyond 1.5 times the interquartile range. P-values by two-tailed, two-sample t-tests with Benjamini–Hochberg correction.

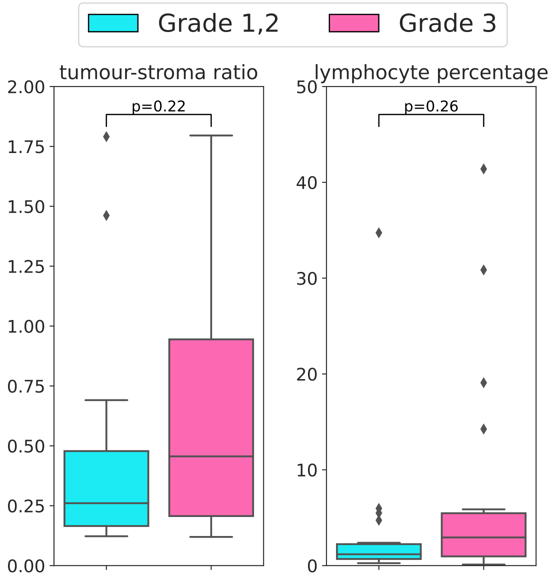

Supplementary Figure 6: Bar plot of the stroma region relative to tumour region for grade 1 and 2 or grade 3 in ER positive patients, with outliers defined as values beyond 1.5 times the interquartile range. P-values by two-tailed, two-sample t-tests.

Supplementary Video 1: This video shows relatively normal vessels at the tumour border and the skin, while within the tumour the vessels appear more disorganised.

Supplementary Video 2: Vascular structures at the tumor border and highly disorganised vessels within the tumour.

Supplementary Video 3: Highly disorganised vessels within the tumour, including disconnected fragments and latices.

Supplementary Video 4: This video shows the tumour region near a necrotic area where, in addition to highly disorganised vessels within the tumour, we can see regular vessels within the necrotic area.
